# Induction indices during ventricular pacing as an alternative diagnostic tool in supraventricular tachycardias

**DOI:** 10.1016/j.hroo.2026.03.001

**Published:** 2026-03-09

**Authors:** Akinobu Mizutani, Masato Okada, Mebae Mizutani, Naoko Miyazaki, Koji Tanaka, Yuko Hirao, Kohei Iwasa, Heitaro Watanabe, Yasushi Koyama, Yoshitaka Iwanaga, Atsunori Okamura, Katsuomi Iwakura, Yasushi Sakata, Nobuaki Tanaka

**Affiliations:** 1 Cardiovascular Center, Sakurabashi Watanabe Advanced Healthcare Hospital, Osaka, Japan; 2 Department of Cardiovascular Medicine, Osaka University Graduate School of Medicine, Osaka, Japan

**Keywords:** Catheter ablation, Electrophysiology study, Supraventricular tachycardia, Pacing maneuvers, Postpacing interval

## Abstract

**Background:**

Pacing maneuvers are fundamental for differentiating supraventricular tachycardia (SVT) mechanisms, but interpretation may be limited when tachycardia cannot be sustained.

**Objective:**

This study aimed to determine whether SVT induction indices during ventricular pacing (VP) provide diagnostic information comparable with conventional ventricular overdrive pacing (VOP).

**Methods:**

We retrospectively analyzed 92 SVTs (91 patients) induced by VP: 10 atrial tachycardias (ATs), 41 atrioventricular nodal reentrant tachycardias (AVNRTs), and 41 orthodromic reciprocating tachycardias (ORTs). The induction ventriculoatrial sequence and induction postpacing interval (iPPI), defined as the interval from the last VP stimulus to the first ventricular electrogram of induced SVT, were assessed. The difference between the iPPI and tachycardia cycle length (iPPI–TCL) was compared with the conventional postpacing interval (PPI)–TCL in cases successfully entrained by VOP.

**Results:**

All ATs were induced with a ventricular–atrial–atrial–ventricular sequence, whereas all AVNRT and ORT exhibited a ventricular–atrial–ventricular sequence, fully concordant with post-VOP sequences. The iPPI–TCL was significantly longer in AVNRT than ORT (180 [150, 201] vs 80 ms [50, 129]; *P* < .001) and correlated with the PPI–TCL (r = 0.685; *P* < .001). After atrial–His correction, the corrected iPPI–TCL strongly correlated with the corrected PPI–TCL (r = 0.948; *P* < .001). A corrected iPPI–TCL cutoff of ≤110 ms differentiated ORT from AVNRT with 90.2% sensitivity and 100% specificity and identified septal ORT without misclassification.

**Conclusion:**

The induction ventriculoatrial sequence reliably differentiated AT from AVNRT/ORT, and the iPPI–TCL closely paralleled conventional PPI–TCL. Induction indices during VP may serve as practical alternatives when PPI measurements are not feasible.

## Graphical abstract

**Figure:**
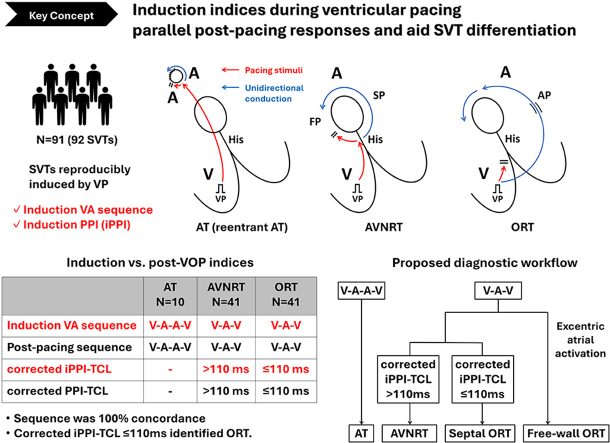

**Key Findings**

▪ The induction ventricular–atrial sequence distinguished atrial tachycardia (ventricular–atrial–atrial–ventricular) from atrioventricular nodal reentrant tachycardia (AVNRT) or orthodromic reciprocating tachycardia (ORT) (ventricular–atrial–ventricular).
▪ The induction postpacing interval minus tachycardia cycle length (iPPI–TCL) paralleled the conventional postpacing interval minus TCL, and a corrected iPPI–TCL of ≤110 ms differentiated septal ORT from AVNRT.
▪ Combining these induction-based indices enhances differentiation of supraventricular tachycardia mechanisms when tachycardia is too brief for entrainment.

## Introduction

Accurate identification of supraventricular tachycardia (SVT) mechanisms is essential for guiding catheter ablation and avoiding unnecessary lesions. Diagnostic pacing maneuvers during SVT, particularly ventricular overdrive pacing (VOP), play a pivotal role in this process, allowing both analysis of the atrial response and assessment of the postpacing interval minus tachycardia cycle length (PPI–TCL).^1^, ^2^, ^3^, ^4^, ^5^ A ventricular–atrial–ventricular (V-A-V) response strongly favors atrioventricular nodal reentrant tachycardia (AVNRT) or orthodromic reciprocating tachycardia (ORT), whereas a ventricular–atrial–atrial–ventricular (V-A-A-V) response indicates atrial tachycardia (AT).^1^ Similarly, a shorter PPI−TCL from the right ventricular apex supports ORT over AVNRT.^2^^,^^3^

Despite its utility, successful VOP may be hindered by TCL oscillations, failure to entrain, and tachycardia termination during pacing. Although termination analysis during VOP can provide additional diagnostic clues,^6^ differential diagnosis remains challenging when tachycardia is poorly sustained.

A previous study suggested that the ventriculoatrial (VA) response immediately after tachycardia initiation parallels the physiology observed during VOP; however, this concept was evaluated only in atypical AVNRT and ORT and was not tested across the full SVT spectrum.^7^ This raises the question of whether induction pacing maneuvers can yield diagnostic insights analogous to those obtained during VOP.

Therefore, we evaluated 2 induction indices obtained during ventricular pacing (VP): (1) the induction VA (iVA) sequence (V-A-V or V-A-A-V) and (2) the induction postpacing interval (iPPI). We then examined whether these induction indices could serve as practical alternatives to conventional postpacing measurements after cessation of VOP.

## Methods

### Study population

This single-center observational study evaluated 746 consecutive patients who underwent an initial electrophysiological study and catheter ablation for SVT at Sakurabashi Watanabe Hospital, Osaka, Japan, between 2015 and 2024. After review of ablation reports and confirmation of catheter laboratory data, 92 SVTs (91 patients) met the following inclusion criteria: (1) reproducible induction by VP and (2) availability of complete intracardiac electrogram recordings for offline analysis. Reproducible induction was defined as induction of the same SVT on at least 2 separate attempts using VP, with a consistent intracardiac atrial activation sequence and no apparent pacing–tachycardia fusion on the atrial electrograms. SVT mechanisms were diagnosed according to established electrophysiological criteria and confirmed by successful ablation.^5^ A written informed consent for ablation and anonymized data use was obtained from all participants. The research reported in this paper was approved by the institutional review board (SWH #25-13) and adhered to the Declaration of Helsinki.

### Electrophysiological study protocol

After vascular access was obtained, standard quadripolar catheters were positioned in the high right atrium, His-bundle region, coronary sinus, and right ventricular apex. The 12-lead surface electrocardiograms and intracardiac electrograms were recorded and stored digitally; intracardiac signals were filtered with a bandpass of 30–500 Hz.

At baseline, VP from the right ventricular apex was routinely performed early during the electrophysiological study to assess retrograde VA conduction. Ventricular burst pacing (cycle length 600–300 ms) was used to determine the VA Wenckebach and/or 2:1 VA block rate. Programmed ventricular stimulation was then performed (basic cycle length 600 ms; S1–S2 shortened by 10 ms to the ventricular effective refractory period or 200 ms). Subsequently, atrial burst and extrastimulus pacing were performed similarly to assess antegrade conduction and induce SVT. If SVT was not inducible at baseline, these maneuvers were repeated during isoproterenol infusion.

Once an SVT was induced and sustained, VOP was performed at a cycle length of 10–40 ms shorter than the TCL. Entrainment was confirmed by acceleration of the atrial rate to the pacing cycle length without a change in the atrial activation sequence, followed by resumption of the original tachycardia after pacing cessation.

### Diagnosis of the SVT mechanism

#### AT diagnosis

AT was diagnosed if ventricular activation was not linked to atrial activation, demonstrated by at least 1 of the following: (1) a delta VA interval of >20 ms after cessation of differential atrial overdrive pacing^8^; (2) an A–H–V activation sequence after cessation of atrial overdrive pacing in the presence of orthodromic atrial activation, in which the captured atrial electrogram propagated in an orthodromic fashion^9^; or (3) a V-A-A-V response after VOP together with a markedly prolonged interval between the last captured atrial electrogram and the first atrial beat of the tachycardia, substantially exceeding the TCL.^1^ AT was excluded if any of the following were observed: a V-A-V response after VOP, tachycardia termination without atrial capture during VOP or premature ventricular contraction; prolongation of the atrial cycle length in response to premature ventricular contraction, tachycardia termination after a single atrial extrastimulus or spontaneous atrio–Hisian (AH) block,^10^ or the presence of VA block during tachycardia.

Reentrant AT was operationally defined as AT that was reproducibly inducible and terminable by programmed stimulation and demonstrated manifest entrainment by atrial overdrive pacing from at least 1 atrial site when assessed.^6^^,^^9^ In cases in which manifest entrainment could not be clearly demonstrated, the mechanism was classified as undetermined.

#### ORT diagnosis

ORT was diagnosed if 1 or more of the following criteria were met: (1) PPI–TCL of ≤115 ms^2^ or corrected PPI–TCL of ≤110 ms^3^; (2) orthodromic His capture during VOP, or prolongation of TCL without AH prolongation during ipsilateral bundle branch block of the accessory pathway; (3) SA–VA interval of <85 ms^2^; (4) constant or progressive fusion during VOP; or (5) total pacing prematurity of <125 ms.^6^ ORT was excluded in the presence of AH block during tachycardia or antidromic His capture without corresponding TCL capture during VOP or a premature ventricular contraction.

#### AVNRT diagnosis

AVNRT was diagnosed when the findings were inconsistent with both AT and ORT. Subclassification was based on the conduction pathway characteristics: an AH interval of >200 ms indicated anterograde conduction via a slow pathway, whereas a retrograde His–atrial interval of >70 ms indicated a slow retrograde limb. The earliest atrial activation site (EAAS) was also used to define subtypes as slow–fast, fast–slow, or slow–slow, according to whether the EAAS was located near the His-bundle region or proximal coronary sinus.

### Induction indices during VP

**Figure 1:**
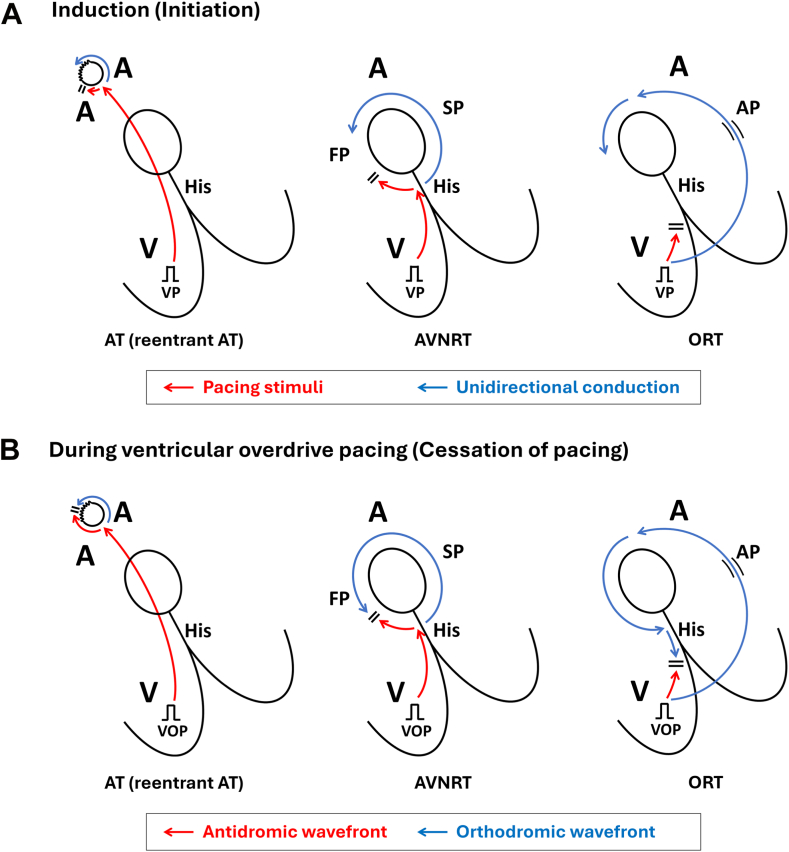
Theoretical basis of induction indices during ventricular pacing. **A:** During induction, the interval from the last ventricular pacing stimulus to the first ventricular electrogram of tachycardia (induction postpacing interval) represents conduction from the pacing site to the tachycardia circuit, 1 rotation within the circuit, and return conduction to the ventricle. A functional block in 1 limb of the dual pathways and unidirectional conduction allows the initiation of reentry. **B:** During ventricular overdrive pacing, the orthodromic (N) and antidromic (N–1) wavefronts continuously reset the circuit, creating a stable state. After pacing cessation, the postpacing interval is measured, reflecting conduction through the same components during entrainment. Thus, induction postpacing interval–tachycardia cycle length conceptually parallels postpacing interval–tachycardia cycle length as an estimate of extracircuit conduction time. AT = atrial tachycardia; AVN = atrioventricular node; AVNRT = atrioventricular nodal reentrant tachycardia; FP = fast pathway; ORT = orthodromic reciprocating tachycardia; SP = slow pathway; VOP = ventricular overdrive pacing; VP = ventricular pacing.

**Figure 2:**
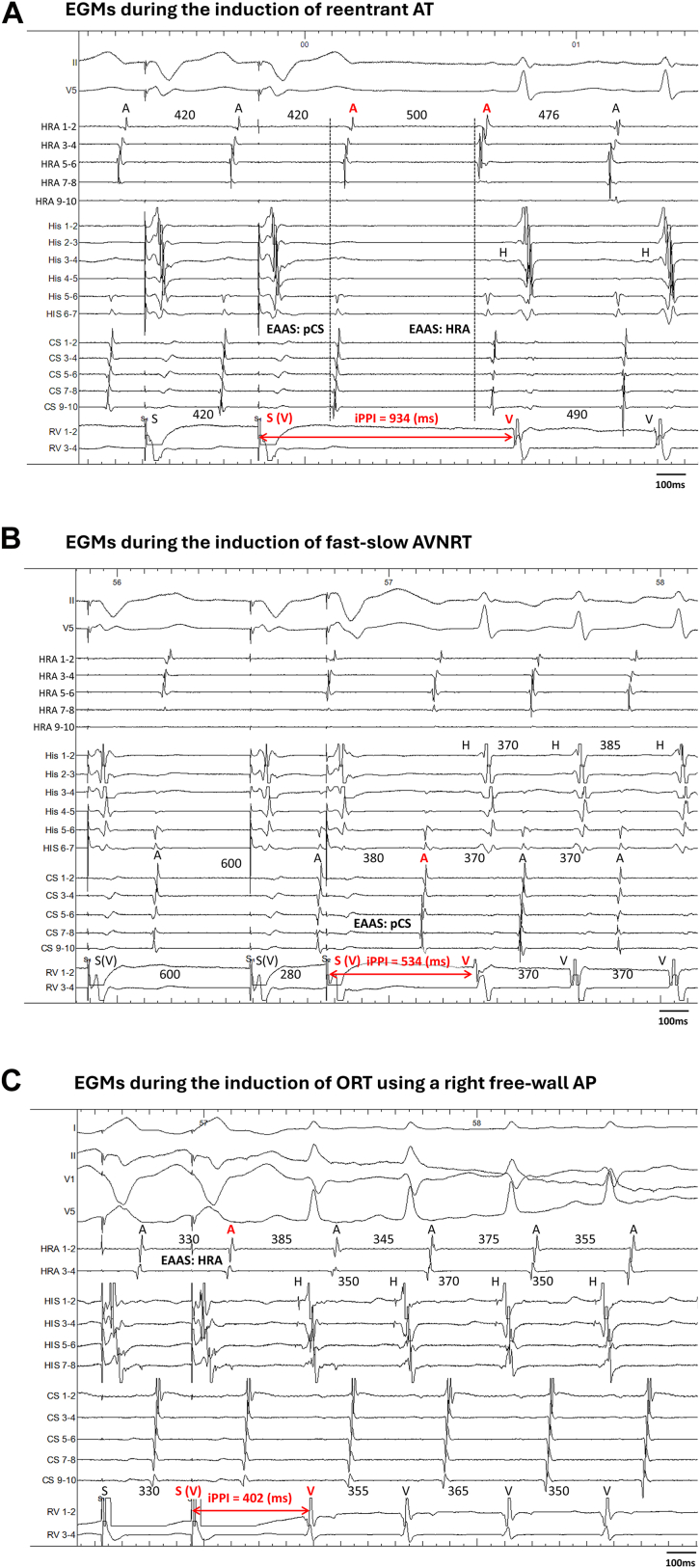
Intracardiac EGMs during induction of supraventricular tachycardias. **A:** AT induced by ventricular burst pacing with a ventricular–atrial–atrial–ventricular sequence; the EAAS shifted from the CS ostium during ventricular pacing to the HRA during the AT (iPPI 934 ms). **B:** Fast–slow AVNRT induced by ventricular extrastimuli with a ventricular–atrial–ventricular sequence; the EAAS remained at the CS ostium (iPPI 534 ms). **C:** ORT using a right free-wall accessory pathway induced by ventricular extrastimuli with a ventricular–atrial–ventricular sequence; the EAAS remained at the CS ostium (iPPI 402 ms). AT = atrial tachycardia; AVNRT = atrioventricular nodal reentrant tachycardia; CS = coronary sinus; EAAS = earliest atrial activation site; EGM = electrogram; HRA = high right atrium; iPPI = induction postpacing interval; ORT = orthodromic reciprocating tachycardia.

**Figure 3:**
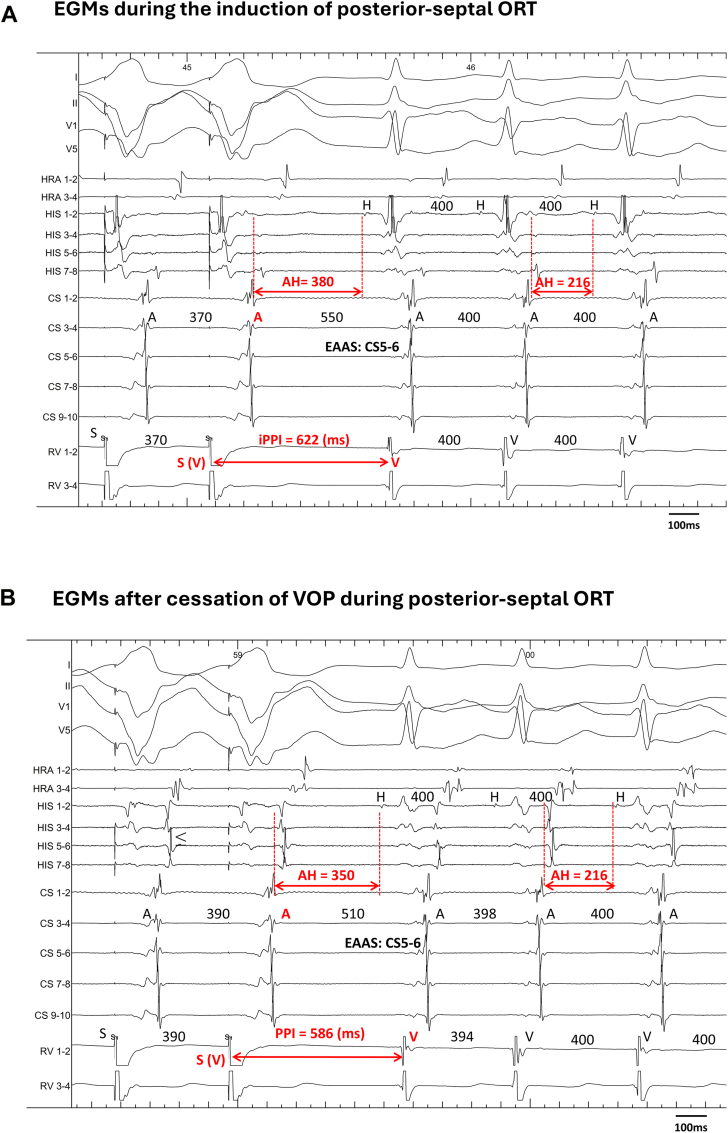
Influence of AH prolongation on the iPPI measurement. Intracardiac EGMs during the induction of posterior-septal ORT showing marked prolongation of iPPI–TCL owing to atrioventricular nodal decremental conduction. **A:** Septal ORT induced by ventricular extrastimuli with a ventricular–atrial–ventricular sequence; the EAAS was recorded on the CS 5–6. The iPPI and iPPI–TCL were 622 and 222 ms, respectively. The AH interval at initiation was longer than that during tachycardia (380 vs 216 ms). **B:** VOP during the ORT. After pacing cessation, tachycardia resumed with a ventricular–atrial–ventricular sequence; PPI was 586 ms. Although the PPI–TCL was 186 ms, AH correction reduced the corrected PPI–TCL to 52 ms. Applying the same correction, the corrected iPPI–TCL was 86 ms. AV = atrioventricular; EAAS = earliest atrial activation site; EGM = electrogram; iPPI = induction postpacing interval; PPI = postpacing interval; TCL = tachycardia cycle length; VOP = ventricular overdrive pacing.

During SVT induction, the paced wavefront propagates from the pacing site to the tachycardia circuit, completes 1 full rotation, and returns to the ventricle. This conduction pattern is conceptually analogous to that observed after cessation of pacing during entrainment (Figure 1).^4^ Unlike previous studies that primarily analyzed VOP responses,^4^^,^^6^^,^^9^^,^^11^ the present study focused on the initiation phase and evaluated the following induction indices:

1. iVA sequence: the atrial–ventricular activation sequence observed during SVT initiation induced by either ventricular extrastimuli or ventricular burst pacing, categorized as V-A-V or V-A-A-V. The initial V was defined as the last pacing stimulus, and the return V as the first ventricular activation recorded on the right ventricular apical pacing catheter after cessation of pacing. The initial A was defined as the earliest atrial electrogram of the last entrained atrial activation. A V-A-A-V response was defined as 2 discrete atrial activations after the last paced beat. His-potential timing was incorporated into sequence adjudication to avoid misclassifying pseudo V-A-A-V responses owing to delayed local His-region ventricular electrograms.^12^
2. iPPI: the interval from the last VP stimulus to the first ventricular electrogram of the induced SVT. For analysis, the difference between the iPPI and the tachycardia cycle length (iPPI–TCL) was calculated. In patients with successful entrainment by VOP with 1:1 VA conduction, the conventional PPI–TCL was also measured for direct comparison with the iPPI–TCL. Representative examples of the iVA sequences and iPPI–TCL measurements are presented in Figure 2. To account for decremental prolongation of the AH interval, corrected iPPI–TCL was calculated as iPPI–TCL minus the AH difference between induction and stable tachycardia (Figure 3), analogous to the corrected PPI–TCL in SVTs with a V-A-V response.

### Statistical analysis

Continuous variables were expressed as median (interquartile range) and compared using the Mann–Whitney U test or the Kruskal–Wallis test, given the non-normal distributions of the measurements. Categorical variables were expressed as numbers (%) and were analyzed using the χ^2^ test or Fisher’s exact test, as appropriate.

The analysis proceeded as follows. First, iPPI–TCL and corrected iPPI–TCL were summarized across the entire cohort of 92 SVTs. Second, in cases of successful VOP with 1:1 VA conduction, concordance of the VA sequence during induction and after cessation of pacing was assessed. Third, in AVNRT and ORT, iPPI–TCL and corrected iPPI–TCL were compared with conventional PPI–TCL and corrected PPI–TCL, respectively. Correlations between induction and conventional indices were assessed using Spearman’s rank correlation coefficient. Fourth, the diagnostic performance of iPPI–TCL and corrected iPPI–TCL in distinguishing AVNRT from ORT was evaluated in the overall cohort using receiver operating characteristic curve (ROC) analysis. Areas under the curve (AUCs) with 95% confidence intervals were calculated, with the optimal cutoff values determined using the Youden index. As an exploratory analysis, iPPI–TCL and corrected iPPI–TCL were also compared between AVNRT and septal ORT.

All analyses were performed on a complete-case basis, without imputation of missing data. Statistical analyses were performed using MedCalc (MedCalc Software, Ostend, Belgium) and R version 4.4.2 software (R Foundation for Statistical Computing, Vienna, Austria). A 2-sided *P* < .05 was considered statistically significant.

## Results

### Study population

Baseline characteristics and VOP findings are presented in Table 1. The cohort included 10 ATs, 41 AVNRTs, and 41 ORTs. Female sex was more prevalent in AT and AVNRT than in ORT (80%, 73%, and 17%, respectively; *P* < .001). SVT was induced by ventricular burst pacing in 61 cases and by ventricular extrastimuli in 31 cases, with no significant difference among mechanisms. Of the 10 ATs, 2 originated near the sinus node, 1 from the crista terminalis, 1 from the vicinity of the atrioventricular node, and 6 from other tricuspid annular regions. Among the 41 AVNRTs, 18 were slow–fast, 16 were fast–slow, and 7 were slow–slow. Of the 41 ORTs, 35 involved a free-wall accessory pathway (34 left free wall and 1 right free wall), and 6 involved a septal accessory pathway (1 anterior and 5 posterior).

**Table 1:** Baseline characteristics and VOP findings

Characteristics	AT (n = 10)	AVNRT (n = 41)	ORT (n = 41)	*P* value
Age, y	70 (58, 74)∗	65 (56, 75)∗	53 (35, 65)†^,^‡	<.001
Female, n (%)	8 (80)	30 (73)	7 (17)	<.001
Induction VP method, n (%)				
Extrastimuli	5 (50)	12 (29)	14 (34)	.46
Burst pacing	5 (50)	29 (71)	27 (66)	
Type of tachycardia	Reentrant: 8 (80) Undetermined: 2 (20)	Slow–fast: 18 (44) Fast–slow: 16 (39) Slow–slow: 7 (17)	Septal: 6 (15) Free wall: 35 (85)	-
TCL, ms	405 (385, 470)∗	390 (354, 433)∗	365 (344, 401)†^,^‡	.151
SA–VA interval	-	144 (120, 165)∗	71 (56, 100)‡	<.001
Responses to VOP, n (%)				
Termination	2 (20)	3 (7)	12 (29)	.037
Successful entrainment	8 (80)	38 (93)	29 (71)	
Post-VOP sequence§				
V-A-V	0/8 (0)	38/38 (100)	29/29 (100)	<.001
V-A-A-V	8/8 (100)	0/38 (0)	0/29 (0)	
Post-VOP indices||				
PPI–TCL, ms	-	165 (150, 180)∗	120 (90, 156)‡	<.001
Corrected PPI–TCL, ms	-	150 (135, 160)∗	90 (61, 100)‡	<.001

AT = atrial tachycardia; AVNRT = atrioventricular nodal reentrant tachycardia; ORT = orthodromic reciprocating tachycardia; PPI = postpacing interval; SA = stimulus-atrial; TCL = tachycardia cycle length; VA = ventriculoatrial; V-A-V = ventricular–atrial–ventricular; V-A-A-V = ventricular–atrial–atrial–ventricular; VOP = ventricular overdrive pacing; VP = ventricular pacing.

∗ *P* < .05 vs ORT.

† *P* < .05 vs AT.

‡ *P* < .05 vs AVNRT.

§ Postpacing sequences were assessed only after successful entrainment with a 1:1 VA relationship and a fixed VA interval during VOP.

|| Postpacing indices are examined in AVNRT and ORT cases.

During VOP, 17 SVTs were terminated, whereas 75 (81.5%) were successfully entrained with 1:1 VA conduction. Among the 75 entrained SVTs, all entrained ATs exhibited a V-A-A-V activation sequence, whereas all AVNRTs and ORTs exhibited a V-A-V sequence after cessation of VOP. The PPI–TCL was significantly shorter in ORTs than in AVNRTs (120 ms [90–156] vs 165 ms [150–180]; *P* < .001). However, the median value in ORTs was above the conventional cutoff of 115 ms (≤115 ms supporting ORT over AVNRT). After AH correction, the median corrected PPI–TCL in ORTs was 90 ms, which was within the conventional corrected cutoff of 110 ms (≤110 ms supporting ORT over AVNRT).

### Analysis of the iVA sequence

During VP, all AVNRTs (n = 41) and ORTs (n = 41) were induced with a V-A-V sequence, whereas all ATs (n = 10) were initiated with a V-A-A-V sequence. Among the 75 SVTs successfully entrained with 1:1 VA conduction, the iVA sequence was fully concordant with that observed after pacing cessation (100% concordance).

### Analysis of the iPPI

**Figure 4:**
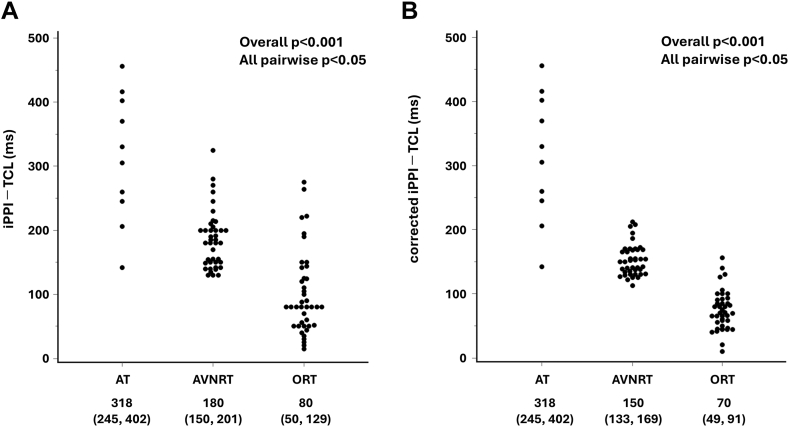
Comparison of induction ventricular intervals across SVTs. Dot plots of iPPI–TCL (A) and corrected iPPI–TCL (B) in AT, AVNRT, and ORT. AH correction was applied only to AVNRT and ORT with a ventricular–atrial–ventricular response. Differences were significant overall (*P* < .001) and for all pairwise comparisons (*P* < .05). AT = atrial tachycardia; AVNRT = atrioventricular nodal reentrant tachycardia; iPPI = induction postpacing interval; ORT = orthodromic reciprocating tachycardia; TCL = tachycardia cycle length.

The median iPPI differed among mechanisms (AT 740 ms [630, 825]; AVNRT 570 ms [530, 645]; ORT 460 ms [408, 527]; *P* < .001; all pairwise *P* < .05). Consistently, iPPI–TCL also differed significantly across SVTs (AT 318 ms [245–402]; AVNRT 180 ms [150–201]; ORT 80 ms [50–129]) (Figure 4A) and paralleled PPI–TCL (Supplemental Figure 1). After AH correction in AVNRT and ORT, the results remained consistent with reduced variability (Figure 4B).

### Correlation between iPPI–TCL and PPI–TCL

**Figure 5:**
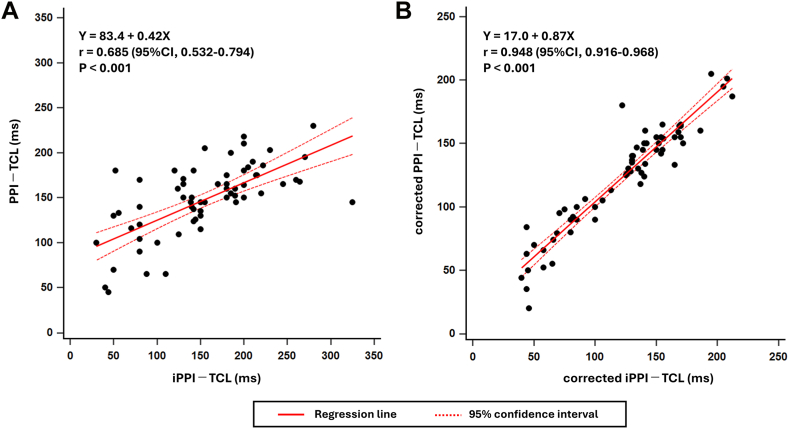
Correlation between iPPI–TCL and PPI–TCL. Scatter plots in 67 entrained AVNRT/ORT cases with regression lines and 95% CIs: iPPI–TCL vs PPI–TCL (r = 0.685; *P* < .001) (A) and corrected iPPI–TCL vs corrected PPI–TCL (r = 0.948; *P* < .001) (B). AVNRT = atrioventricular nodal reentrant tachycardia; CI = confidence interval; iPPI = induction postpacing interval; ORT = orthodromic reciprocating tachycardia; PPI = postpacing interval; TCL = tachycardia cycle length.

In 67 AVNRT/ORT cases with successful PPI measurements, iPPI–TCL and PPI–TCL were moderately correlated (Spearman r = 0.685; *P* < .001). After AH correction, the corrected iPPI–TCL and corrected PPI–TCL were strongly correlated (r = 0.948; *P* < .001) (Figure 5).

### Diagnostic accuracy of the iPPI–TCL for ORT

**Figure 6:**
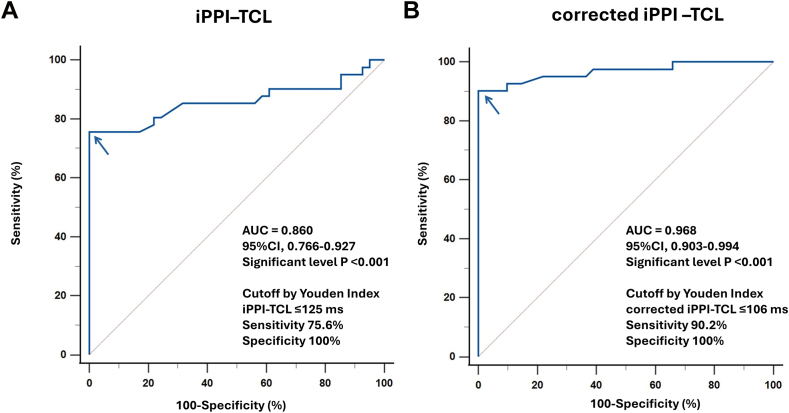
Diagnostic performance of iPPI–TCL for ORT. Receiver operating characteristic curves for iPPI–TCL (A) and corrected iPPI–TCL (B) to differentiate ORT from AVNRT. AUCs are shown; optimal cutoffs were determined by the Youden index. AUC = area under the curve; AVNRT = atrioventricular nodal reentrant tachycardia; iPPI = induction postpacing interval; ORT = orthodromic reciprocating tachycardia; TCL = tachycardia cycle length.

ROC analysis showed that the iPPI–TCL effectively distinguished ORT (n = 41) from AVNRT (n = 41), with an AUC of 0.860 (*P* < .001). A cutoff of ≤125 ms provided 100% sensitivity and 75.6% specificity, with a positive predictive value of 80.4% and a negative predictive value of 100% (Figure 6A). After AH correction, the corrected iPPI–TCL demonstrated an improved AUC of 0.968, and the conventional postpacing cutoff of ≤110 ms yielded 90.2% sensitivity and 100% specificity, with a positive predictive value of 100% and a negative predictive value of 91.1% in this cohort (ORT prevalence 50%). An ROC-derived cutoff of ≤106 ms showed identical diagnostic performance (90.2% sensitivity and 100% specificity) (Figure 6B).

**Figure 7:**
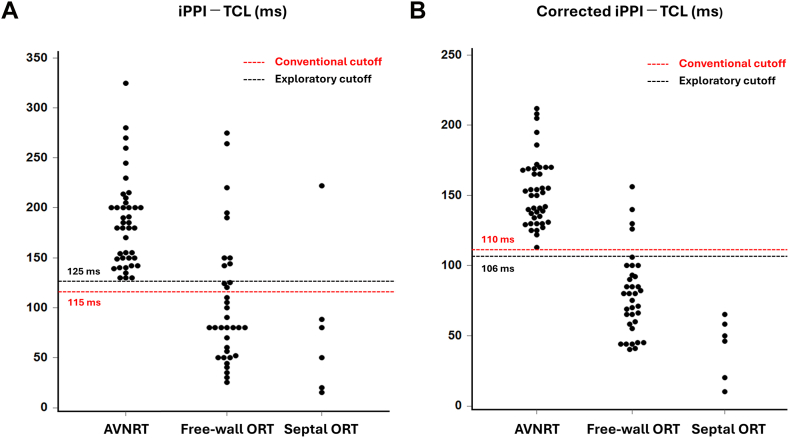
Diagnostic separation between AVNRT and septal ORT. Dot plots of iPPI–TCL (A) and corrected iPPI–TCL excluding AT (B) (n = 10). When ORTs were subclassified into free-wall (n = 35) and septal ORT (n = 6), iPPI–TCL largely separated AVNRT from septal ORT, with 1 overlapping case, whereas corrected iPPI–TCL exhibited complete separation. Using the conventional PPI–TCL thresholds as references, iPPI–TCL of ≤115 ms and corrected iPPI–TCL of ≤110 ms identified septal ORT with sensitivities/specificities of 83.3%/100% and 100%/100%, respectively. Exploratory cutoffs (iPPI–TCL ≤125 ms; corrected iPPI–TCL ≤106 ms) performed similarly in this cohort. AT = atrial tachycardia; AVNRT = atrioventricular nodal reentrant tachycardia; iPPI = induction postpacing interval; ORT = orthodromic reciprocating tachycardia; PPI = postpacing interval; TCL = tachycardia cycle length.

When analysis was restricted to patients with septal ORT (n = 6), the iPPI–TCL showed near-complete separation between AVNRT and septal ORT, with 1 septal ORT case overlapping the AVNRT distribution (Figure 7). Using the conventional PPI–TCL threshold as a reference, iPPI–TCL of ≤115 ms identified ORT with 83.3% sensitivity and 100% specificity. After AH correction, the corrected iPPI–TCL was completely separated in this sample; the conventional cutoff of ≤110 ms identified ORT without misclassification. An exploratory subgroup analysis comparing typical slow–fast AVNRT (n = 18) with septal ORT is presented in Supplemental Figure 2. Although limited by sample size, the results were consistent with the main analysis and are presented descriptively.

## Discussion

The diagnostic concordance between induction indices and entrainment-based measurements has been demonstrated in AVNRT and ORT.^7^ Our findings extend this concept across the full SVT spectrum, including AT, and show that the iVA sequence and iPPI–TCL aid differentiation of SVT mechanisms. The iVA sequence (V-A-A-V vs V-A-V) reliably distinguished AT from AVNRT and ORT. In AVNRT and ORT, iPPI–TCL was moderately correlated with PPI–TCL, and AH correction further strengthened this relationship. Using the conventional PPI–TCL thresholds, the iPPI–TCL identified ORT with high sensitivity and specificity. These findings suggest that VP-based induction indices may serve as practical alternatives to VOP-based assessments, particularly when SVT is not sustained and entrainment is not feasible.

### Interpretation of the iVA sequence

The iVA sequence during VP paralleled the post-VOP responses: AT consistently exhibited a V-A-A-V sequence, whereas AVNRT and ORT demonstrated a V-A-V sequence. This concordance suggests that the induction sequence reflects the same wavefront conduction dynamics as the postpacing response. In AVNRT and ORT, retrograde conduction from the ventricle activates the atrium and then reactivates the ventricle, producing a V-A-V pattern. Conversely, in AT (particularly reentrant AT), retrograde conduction must traverse a slow atrial pathway before ventricular capture, yielding a V-A-A-V sequence.

A potential consideration is that a V-A-A-V response may also be observed in fast–slow AVNRT, typically owing to a double atrial response.^13^, ^14^, ^15^ For differentiation, assessment of the A-A interval may be informative: the A-A interval is generally longer in reentrant AT than in AVNRT with double atrial responses,^15^ and a longer V-V interval (or iPPI) may further support discrimination. In addition, in slow–fast AVNRT, delayed ventricular electrograms may also yield an apparent V-A-A-V or “pseudo A-A-V” sequence after cessation of VOP; incorporating His-bundle timing may help avoid misclassification.^12^ Although such cases were not observed in our cohort, these considerations indicate that a V-A-A-V sequence is not specific for AT. Larger studies, including diverse AVNRT subtypes,^15^^,^^16^ will be needed to clarify these distinctions.

### Interpretation of the iPPI–TCL

#### Physiological basis

The iPPI represents conduction from the pacing site to the tachycardia circuit, 1 rotation through the circuit, and return conduction to the ventricle—a sequence conceptually analogous to the PPI (Figure 1). Accordingly, the iPPI–TCL mirrors the same “extra-circuit” conduction time estimated by the PPI–TCL. In AVNRT, this interval is prolonged because the paced wavefront must reach and traverse atrioventricular nodal pathways. In ORT, involvement of ventricular myocardium within the circuit shortens this interval. In reentrant AT, although not formally evaluated in this study, the interval may be further prolonged because the paced wavefront must reach the atrium, traverse the atrial reentrant circuit, and then return to the ventricle (Supplemental Figure 1). These pathways are the same routes traversed by the last paced wavefront after VOP cessation, which likely explains the observed correlation between the iPPI–TCL and PPI–TCL.

Despite this mechanistic analogy, the iPPI–TCL and PPI–TCL are not identical. As shown in Figure 5, iPPI was often longer than the PPI, which may reflect transient induction-phase conditions. Reentry initiation typically requires a functional block in 1 limb and conduction slowing in another, which may prolong the iPPI. In contrast, the PPI–TCL is measured during VOP with stable 1:1 entrainment, when conduction properties are more stable.

#### AH correction and diagnostic thresholds

AH correction substantially improved the agreement between iPPI–TCL and PPI–TCL; however, the 2 indices were not identical. This is expected because AH correction adjusts only anterograde atrioventricular nodal delay and cannot account for induction-phase variability in the retrograde limb, including early postinduction VA fluctuations.^17^ This limitation may be more pronounced in accessory pathways with retrograde decremental properties, which can further delay VA conduction and prolong iPPI-derived indices even after AH correction. Nonetheless, AH correction remains a practical adjustment to reduce overestimation of iPPI–TCL. Notably, 1 case of septal ORT exhibited an apparently prolonged iPPI–TCL exceeding the 115-ms postpacing cutoff; however, the value normalized to <100 ms after correction, consistent with the true diagnosis (Figures 3 and 7).

Importantly, the ROC-derived optimal cutoffs for corrected iPPI–TCL (115 ms in a previous study^7^ and 106 ms in our study) closely aligned with the established 110-ms threshold used for corrected PPI–TCL.^3^ The modest differences likely reflect physiological and methodological variability (pacing protocol, sample size, and intrinsic conduction variability) rather than a fundamental difference between iPPI- and PPI-derived indices. The close agreement between these thresholds further supports the concept that corrected iPPI–TCL captures the same physiological construct as corrected PPI–TCL. Taken together, these observations indicate that corrected iPPI–TCL offers robust diagnostic separation between AVNRT and septal ORT and serves as a practical alternative when conventional PPI measurements are not feasible.

### Clinical implications

When SVT is too brief to permit entrainment, the iVA sequence and iPPI–TCL may serve as useful alternatives to VOP-based measurements, without requiring additional pacing maneuvers. Because many free-wall ORTs and nonseptal ATs can be identified based on the atrial activation sequence alone, the incremental diagnostic value of VP-based indices is likely greatest in diagnostically challenging scenarios, particularly when distinguishing septal ORT from AVNRT and septal/annular AT. Consistent with this clinical focus, the septal ORT subgroup analysis showed similar performance in this scenario (Figure 7 and Supplemental Figure 2). Although further studies are required, incorporating these indices into a simplified diagnostic flow may be useful in patients with short-lived, unstable, or difficult-to-sustain tachycardias.

### Limitations

Several limitations should be acknowledged. First, this was a single-center retrospective analysis in which cases were identified by screening ablation reports rather than systematic prospective enrollment. Because documentation of VP–induced SVT was operator dependent, the 92/746 screening denominator should not be interpreted as an induction yield; the true frequency may not have been captured precisely. Second, we analyzed only cases with unequivocal recordings, which improved measurement reliability but limited sample size, reduced statistical power, and precluded robust determination of optimal cutoffs for iPPI–TCL or corrected iPPI–TCL. Third, although we used a conventional right ventricular apical electrogram–based definition, sequence classification as V-A-V or V-A-A-V may differ when alternative ventricular references (eg, His-region electrograms) or catheter positions are used; therefore, our findings should be interpreted accordingly. Fourth, iPPI may be influenced by right bundle branch block and catheter position; a functional retrograde right bundle branch block during VOP is not uncommon and may affect PPI values.^18^ In addition, beat-to-beat VA fluctuations early after induction may affect iPPI,^17^ and standardized assessment (eg, averaging several early postinduction beats) may improve reproducibility and warrant evaluation in larger cohorts. Fifth, although our cohort included typical forms of AT, AVNRT, and ORT, other SVT variants (eg, superior slow AVNRT,^16^ bystander accessory pathways,^19^ and other atypical circuits^20^, ^21^, ^22^) were not represented. The applicability of these induction indices to such variants remains uncertain. Finally, VOP during tachycardia is limited by uncertainty regarding whether true entrainment has been achieved. Termination during pacing with immediate reinitiation cannot be excluded. Likewise, when SVT emerges during ventricular burst pacing, it is difficult to determine whether the last paced beat initiated the tachycardia or whether the tachycardia had already begun during the pacing train.

## Conclusion

Induction indices during VP provide diagnostic information comparable with that obtained from entrainment-based measurements. The iVA sequence reliably distinguished AT, and the iPPI–TCL paralleled conventional PPI–TCL. Combining these indices may facilitate differentiation of SVT mechanisms, particularly when tachycardia is not sustained, and may help guide ablation strategies.

## Data availability

The data that support the findings of this study are available from the corresponding author upon reasonable request.

## Declaration of generative AI and AI-assisted technologies in the writing process

The authors used *ChatGPT (OpenAI)* to enhance the English language quality and improve the manuscript’s readability. After using this tool, the authors reviewed and edited the content as needed and take full responsibility for the final content of the manuscript.

## Disclosures

The authors have no conflicts of interest to disclose.

## Footnotes

Appendix Supplementary data associated with this article can be found in the online version at https://doi.org/10.1016/j.hroo.2026.03.001.
