# Supplementary material for: Induction indices during ventricular pacing as an alternative diagnostic tool in supraventricular tachycardias
Source: Heart Rhythm O2. 2026 Mar 9;7(5):861–71. doi: 10.1016/j.hroo.2026.03.001 (PMC13198357; doi:10.1016/j.hroo.2026.03.001)
Supplement: Supplementary Material [file mmc1.docx]

**SUPPLEMENTAL MATERIAL**

**Induction Indices during Ventricular Pacing as an Alternative Diagnostic Tool in Supraventricular Tachycardias**

**Supplemental Figure 1. Comparison of the induction and post-pacing indices**


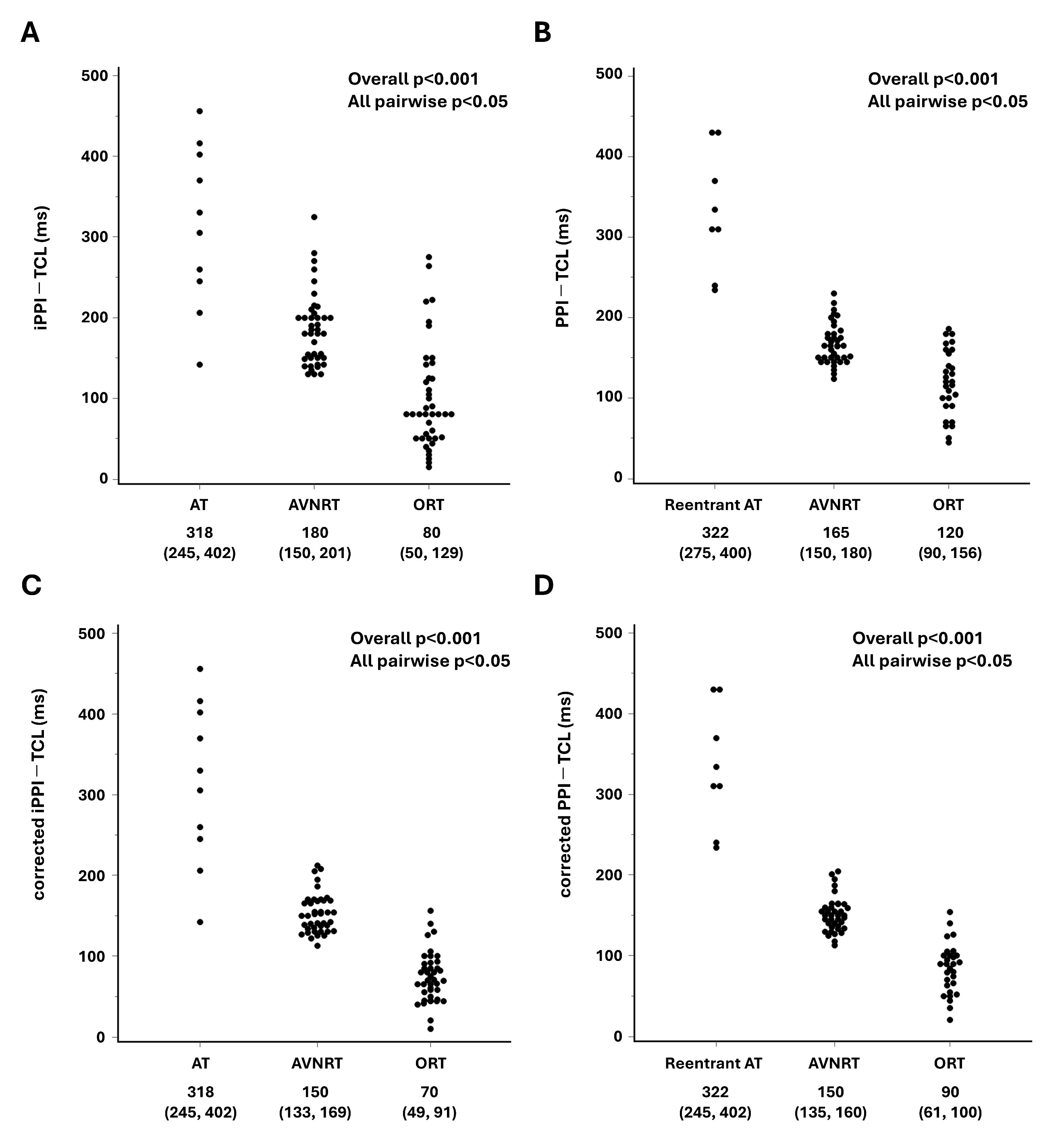


Dot plots showing the induction and post-pacing indices across the three types of supraventricular tachycardia: (A) iPPI–TCL, (B) PPI–TCL, (C) corrected iPPI–TCL, and (D) corrected PPI–TCL. Post-pacing evaluations were performed in AVNRT and ORT, and 8 reentrant ATs that were successfully entrained by VOP with fixed 1:1 conduction and reproducible return intervals. AH correction was applied only in AVNRT and ORT with a V-A-V response. Significant differences were observed among the three groups, with all pairwise comparisons reaching statistical significance (p <0.05).

AT, atrial tachycardia; AVNRT, atrioventricular nodal reentrant tachycardia; iPPI, induction post-pacing interval; ORT, orthodromic reciprocating tachycardia; PPI, post-pacing interval; TCL, tachycardia cycle length.

**Supplemental Figure 2. Comparison of the induction indices between typical AVNRT and septal ORT**

**
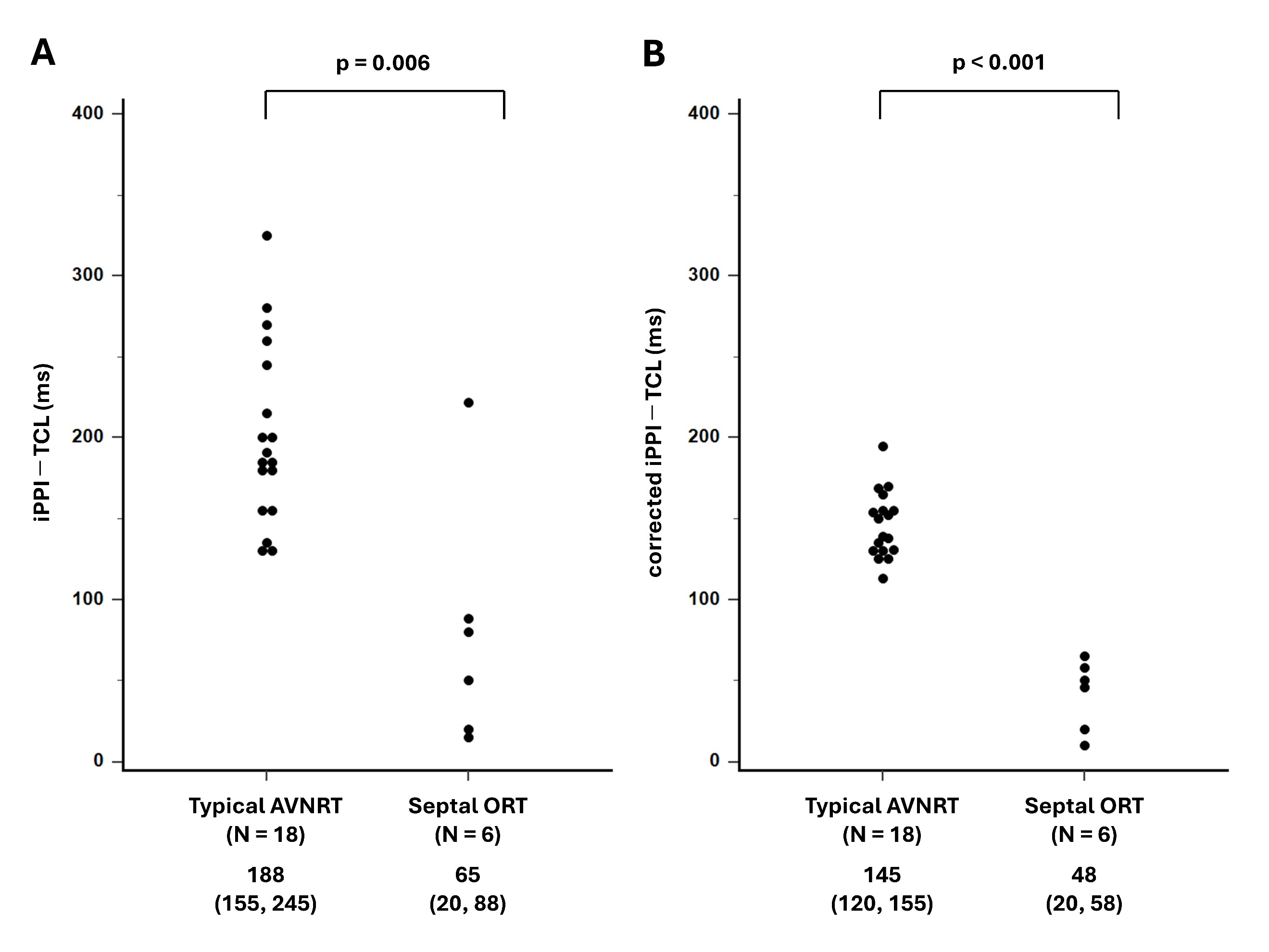
**

Dot plots showing the induction and post-pacing indices between typical AVNRT and septal ORT: (A) iPPI–TCL and (B) corrected iPPI–TCL. The iPPI–TCL showed one overlapping case between typical AVNRT and septal ORT; after AH correction, corrected iPPI–TCL showed complete separation with no observed misclassification in this sample.

AVNRT, atrioventricular nodal reentrant tachycardia; iPPI, induction post-pacing interval; ORT, orthodromic reciprocating tachycardia; TCL, tachycardia cycle length
